# The Effect of Virtual Reality Technology on the Imagery Skills and Performance of Target-Based Sports Athletes

**DOI:** 10.3389/fpsyg.2020.02073

**Published:** 2021-01-22

**Authors:** Deniz Bedir, Süleyman Erim Erhan

**Affiliations:** ^1^Erzurum Technical University, Erzurum, Turkey; ^2^College of Physical Education and Sports, Tekirdağ Namık Kemal Üniversitesi, Tekirdağ, Turkey

**Keywords:** virtual reality, imagery, target sports, visual motor behavior rehearsal, video modeling, PETTLEP, shot performance

## Abstract

The aim of this study is the examination of the effect of virtual reality based imagery (VRBI) training programs on the shot performance and imagery skills of athletes and, and to conduct a comparison with Visual Motor Behavior Rehearsal and Video Modeling (VMBR + VM). In the research, mixed research method and sequential explanatory design were used. In the quantitative dimension of the study the semi-experimental model was used, and in the qualitative dimension the case study design was adopted. The research participants were selected from athletes who were involved in our target sports: curling (*n* = 14), bowling (*n* = 13), and archery (*n* = 7). All participants were randomly assigned to VMBR + VM (*n* = 11), VRBI (*n* = 12), and Control (*n* = 11) groups through the “Research Randomizer” program. The quantitative data of the study was: the weekly shot performance scores of the athletes and the data obtained from the “Movement Imagery Questionnaire-Revised.” The qualitative data was obtained from the data collected from the semi-structured interview guide, which was developed by researchers and field experts. According to the results obtained from the study, there were statistically significant differences between the groups in terms of shot performance and imagery skills. VRBI training athletes showed more improvement in the 4-week period than the athletes in the VMBR + VM group, in terms of both shot performance and imagery skills. In addition, the VRBI group adapted to the imagery training earlier than the VMBR + VM group. As a result, it was seen that they showed faster development in shot performances. From these findings, it can be said that VRBI program is more efficient in terms of shot performance and imagery skills than VMBR + VM, which is the most used imaging training model.

## Introduction

Imagery is the most popular field of research in sports psychology. According to a physical training, it has always attracted the attention of researchers and athletes due to its advantages, such as saving time and energy, being independent of the training environment, and no risk of disability (Jowdy et al., 1989; Ungerleider, 2005; Weinberg and Gould, 2015).

Imagery is the state of creating or re-creating our experiences in our minds, using all our senses (Suinn, 1985; Vangyn et al., 1990; Salmon et al., 1994). More broadly, imagery can be defined as human ability to access previously encoded perceptual information from memory (Kosslyn, 1980; Farah, 1993) to create a complex and sophisticated mental experience of objects, people, or places (Boccia et al., 2019). For effective imagery, the situation in the mind must be experienced with all sensory organs (Roure et al., 1999; Morris et al., 2005; Kehoe and Rice, 2016). Enough vivid, sharp, and clearly imagined situations create very realistic stimuli in our brain (Williams, 1993). As a result, the brain cannot distinguish whether this work is real or a dream, and it gives us the same physiological reactions as if the moment we are feeling in the mind is actually happening in real life (Martens, 1987; Cox, 2007). Thus, skill acquisition time is shortened when physical movement and imagery are combined together (Michalski et al., 2019).

Creating realistic images in the mind is one of the most important criteria that determines the quality of the imagery training. Naturally, the vast majority of research in the field of imagery so far has focused on the fact that an athlete can produce realistic images in his mind. In this context, one of the most widely used methods is the Physical, Environment, Task, Time, Learn, Emotion, Perspective (PETTLEP) approach, which is based on the functional equivalence hypothesis (Finke, 1979; Holmes and Collins, 2001; Smith et al., 2007, 2008; Moran, 2016). The PETTLEP model has been used across a wide range of domains such as sports psychology, cognitive psychology, and neuroscience (Holmes and Collins, 2001). An application that enhances the effect of the PETTLEP model is video modeling (VM). Watching yourself or others is known to have a positive effect on psychological variables such as performance, self-efficacy, and self-regulation (Murphy, 2012). Recent research has focused on the effects of VM on athletes’ sports performance (Munzert et al., 2008; Ste-Marie et al., 2012). The combination of VM and imagery has attracted interest in both neuroscience and sports psychology literature (Rizzolatti and Craighero, 2004). The rationale behind the positive effects associated with VM and imagery is that neural networks (Holmes and Calmels, 2008; Battaglia et al., 2014). Imagery and VM share a number of mental operations and rely on common neural structures (Grezes and Decety, 2001). These structures are similar to some of those active during the preparation, anticipation and in some cases actual production of action. Imagery and VM cause neural responses similar to intracortical and subcortical plasticity that occur during the physical application of a task in the brain (Holmes and Calmels, 2008).

Another popular application developed to increase the effect of imagery is “Visual Motor Behavior Rehearsal (VMBR).” This technique, developed by Suinn, called VMBR, Suinn (1972a, b) combines both imagery and relaxation. Studies have shown that VMBR enhances the effect of imagery (Kolonay, 1977; Noel, 1980; Weinberg et al., 1981, 1982; Hall and Erffmeyer, 1983; Cauraugh and Lidor, 1992; Shipley and Baranski, 2002).

Despite all these developments, mental training is still seen as boring and a waste of time by many athletes (Gould et al., 1999; LeUnes and Nation, 2002; Gould and Maynard, 2009; Weinberg and Gould, 2015). This situation causes athletes and coaches to be biased toward sports psychology. Therefore, there is a need for innovation that can attract the attention of the athletes and shorten the time taken to reach the level of effective imagery. In this context, researchers exploring the possibilities of developing technology (wearable technologies, biomechanics, eagle-eye camera technology, etc.) in order to increase physiological performance, unfortunately did not go far beyond traditional methods in increasing psychological performance for athletes. Researchers have used progressive muscle relaxation (Kim et al., 2011; Boryri et al., 2019; Theologia et al., 2019), video (Smith and Holmes, 2004; Carbone et al., 2020; Meers et al., 2020), and audio (Smith and Holmes, 2004; Sesum and Kajtna, 2018) to enhance the effect of traditional imagery training. Although these innovations increased the effect of the mental training, they did not bring the desired level of success (Craig, 2013; Vignais et al., 2015; Bird, 2019). In this context, new applications are needed that can increase the effect of the imagery training.

In addition, another concept that determines the effect of imagery is perception. Imagery and perception cause similar neural activities in the brain (Dijkstra et al., 2017). If we can create realistic images in our minds, our brain will react as if we really see that image (Maier et al., 2020b). In addition, studies show that there is a positive correlation between perception in visual areas and vividness of imagery. According to these results, we need to enrich the perception in visual areas in order to increase the vividness of imagery (Dijkstra et al., 2017). It seems likely that we will improve our imagery performance by enriching our perception in visual areas. In the imagery training, much more is needed than 2D videos of sports environments, which were previously used to enrich the perception (Helsen and Pauwels, 1993; Williams et al., 1994). For this, there is a need for a virtual world where the athlete can perceive their surroundings actively with their head movements (Craig, 2013).

Virtual reality (VR) technology, which is the most popular product of developing technology today, has the feature to overcome these shortcomings in imagery applications. VR can deceive the human brain’s predictive coding mechanism and create a real feeling of being present in the virtual body and space (Riva et al., 2019). VR has features similar to imagery in terms of the underlying mechanism. The underlying logic behind imagery and VR is to feel an unreal event, time, or environment as if it were actually happening. The most important feature that differentiates VR technology from other applications in the imagery process is that it gives participants the feeling of the experience being real.

In order to increase the sense of reality, desired items can be placed or removed in the VR environment to be created. In this respect, performance tests to be performed in VR environment will be different from classical laboratory tests. Many of the research done in sports psychology (imagery, VMBR, VM, self-talking etc.) tried to determine the pure effect of mental training performed by athletes by limiting external variables. However, sports competitions take place in highly variable and interactive environments. Therefore, measurements made by isolating from the external environment do not always reflect the correct results. The ecological approach, which is an alternative to the traditional one-way research model conducted in this way, advocates including the effects of the environment on the individual (Davaslıgil, 1997). According to the ecological approach, all factors affecting the individual (relationships, environment, social and cultural variables) should be addressed in a research process instead of examining the individual one way (Heft, 2012, 2013). The ecological approach has recently become popular in the field of sports psychology as well as in other fields. In this context, the methods in the researches in the field of psychological skill training started to be designed within the framework of ecological approach (Araujo et al., 2005; Jordet, 2005; Araujo and Kirlik, 2008; Seif-Barghi et al., 2012; Dicks et al., 2015). By integrating VR technology into mental training processes, tests can be organized within the framework of ecological approach. Variables such as type of surface (e.g., grass pitch), the objects involved (e.g., rugby ball) and the events taking place within it (e.g., a set-play) can also be included in the created VR environment (Riley et al., 2009). A virtual environment provides the researcher with an ecologically valid platform for presenting dynamic stimuli in a manner that allows for both the veridical control of laboratory measures and the verisimilitude of naturalistic observation of real life situations (Matheis et al., 2007; Jovanovski et al., 2012). VR environments proffer assessment paradigms that combine the experimental control of laboratory measures with emotionally engaging background narratives to enhance affective experience and social interactions (Parsons, 2015). In the literature review, it is seen that VR has started to be used in ecological approach-based researches in sports psychology (Ya, 2011; Parsons, 2015).

VR is widely used in many fields such as education (Lorenzo et al., 2020; McGarr, 2020; Radianti et al., 2020; Theelen et al., 2020), medicine (Bhattacharjee et al., 2020; Felipe et al., 2020; Maier et al., 2020a; Xin et al., 2020), computer games (Bapka et al., 2018; Yang et al., 2018; Allspaw et al., 2019), new motor skills acquisition (Prasertsakul et al., 2018; Ricca et al., 2018; de Moraes et al., 2020), virtual sights (Errichiello et al., 2019; Trunfio and Campana, 2019; Trunfio et al., 2019). VR studies in sports focused on three areas: performance analysis (Ouadahi et al., 2016; Neumann et al., 2018), simulation development (Pereira et al., 2018) and virtual training (Adamovich et al., 2009; Calabro et al., 2017; Akbas et al., 2019). VR used in the field of sports provides individualized training of technical-tactical as well as motor abilities regardless of the time and place, against a chosen opponent or situation (Akbas et al., 2019). VR minimizes disturbances in complex experiments involving multiple subjects or complex experiment protocols. VR simulation provides accurate control and synchronization of all items in the experiment and reproducibility and comparison among the different trials (Pereira et al., 2018). In addition, due to the immersion and presence in the virtual environment, user participation and motivation can be provided (Slater, 2009). Despite such a wide range of uses and advantage, has not been used with imagery training.

In this context, the aim of the study is to examine the effect of the VRBI training program on the shot performance and imagery skills of the athletes, and to compare it with the popular approach VMBR + VM used in imagery training. The main hypothesis for this study is that VRBI training will contribute more to the shot performance and imagery skills of athletes than VMBR + VM. To test this hypothesis, the athletes in the VRBI and VMBR + VM groups will be compared with the shot performances and imagery skills, as well as interviews with the athletes.

## Materials and Methods

### Research Design

In this study where the imagery skills and shot performance of the athletes are examined, the mixed research method was used. Sequential explanatory design was used in the study. In this design, quantitative data is first collected and analyzed. Then, qualitative data is collected and analyzed to explain the quantitative results in depth (Sözbilir, 2017).

In the quantitative dimension of the research, semi-experimental design with repeat test VMBR + VM, VRBI, and control group was used. Then, a qualitative research design case study was used to examine the data obtained from quantitative results in more depth. In the case study, semi-structured interviews were conducted with the athletes.

### Participants

GPower analysis was performed to calculate the sample size of the athletes who would participate in the research. According to the analysis, a minimum of 33 samples were required in order to have an effect size of 0.25 in 90% power and 95% confidence intervals. In this regard, because of the possibility of some abandoning the process, 39 athletes were included in the study.

According to Ryan and Simons (1981), imagery is more beneficial in sports involving simple cognitive processes and requiring concentration than it is sports involving complex motor skills and cognitive processes (Altıntas and Akalan, 2008). In this context, curling, bowling and archery branches, which have high concentration and simple motor skills and are targeted sports branches, were selected.

A total of 39 participated in the study, but due to various reasons during the training period we worked with 34 athletes from curling (*n* = 14), bowling (*n* = 13), and archery (*n* = 7) branches completed (age = 21, 7 ± 4.33, weekly training time = 7.38 ± 3.59). Participants were divided into random, control (curling = 4, bowling = 4, archery = 3), VMBR + VM (curling = 4, bowling = 5, archery = 2), and VRBI (curling = 6, bowling = 4 Archery = 2) groups through the “Research Randomizer” program. The curling athletes were from the Turkey Curling First League, the bowling athletes were in the Ataturk University Bowling Team, and the archery athletes are from the national leagues. Detailed demographic information of the participants is given in Table 1.

**TABLE 1:** Demographic information of the participants.

**Groups**	**Gender**		**Age (x̄ ± ss)**	**Weekly training time (x̄ ± ss)**
	**Female (n-%)**	**Male (n-%)**		
Control	5-%45,5	6-%54,5	23,81 ± 5,17	7,63 ± 2,80
VMBR + VM	5-%18,2	6-%81,8	20,54 ± 4,76	7,36 ± 4,36
VRBI	7-%58,3	5-%41,7	21,08 ± 2,27	7,16 ± 3,76
Total	17	17	21,79 ± 4.33	7,38 ± 3,59

#### Selection Criteria of Participants

• Not having professional mental training support before working,
• Not having chronic or acute disability problems,
• Being an elite athlete.

### Performance and Scoring Scenarios

Performance scenarios were prepared by people who are experts in their branches and have coaching certificates. Performance scenarios were prepared in different ways for each branch. This included the freeze shot for the curling, the spinning throw for the bowling, and the shot from the distance of 30 m (target of 30 cm × 30 cm, with the classic bow) for the archery.

**FIGURE 1:**
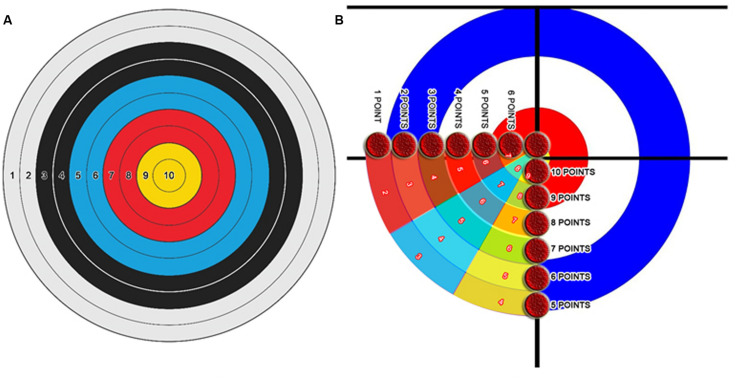
Archery scoring **(A)** and curling scoring **(B)** system.

The prepared performance scenarios were converted to standard scoring by experts and scored over 10 points. The scoring system has been developed for each branch. Bowling athletes were asked to topple 10 pins with a single shot, i.e., to strike. One point is awarded for each topple pin. Archery athletes were asked to shoot at a target of 30 cm × 30 cm from a distance of 30 m with a classic bow. The archery shots are scored according to Figure 1A. Curling athletes were asked to shoot the freeze. The curling shots are scored according to Figure 1B.

### Preparation of Successful Performance Video

**FIGURE 2:**
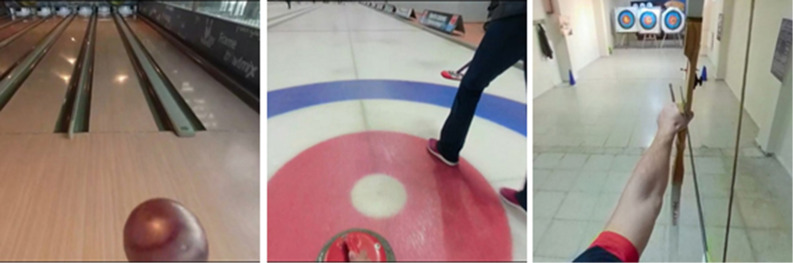
3D performance videos prepared.

The most successful athletes from each branch were selected to play in the shot video to be prepared. In the selection of athletes, being the first player in their teams, the number of national matches, the experience and the opinions of the trainers were determined as criteria. These athletes did not participate in the study. GoPro Fusion 360 camera with high resolution 360-degree video capability was used for performance video shot. The camera was attached to the head of the athlete with an apparatus, to allow the viewing athlete to watch the video from his own perspective and to imagine it from a point of view angle (Figure 2). The successful performance video continued from the locker room to the performance of the athlete. The recorded videos were prepared in 2D video format in computer format for the VMBR + VM group and in 3D video format integrated into the VR glasses for the VRBI group. GoPro Fusion Studio, Adobe Photoshop, Premiere and After Effect programs were used for video formatting.

### Preparation of Progressive Muscle Relaxation Scenario

Progressive Muscle Relaxation Scenario for athletes was prepared with the support of two assistant professors who are experts in sports psychology.

### Measures

#### Movement Imagery Questionnaire-Revised (MIQ-R)

This was developed by Hall et al. (1985) in order to determine the imagery ability of athletes. The scale was revised and simplified by Hall and Martin (1997). The scale consists of eight items that measure visual (four items) and kinesthetic (four items) imagery skills. The scale was adapted to Turkish by Akkarpat (2014).

#### Semi-Structured Interview Form

Face to face interviews were conducted with VMBR + VM and VRBI groups to better explain the quantitative data obtained in the study. At the beginning of the research process, the athletes were informed about the purpose of the training and they were reminded that their participation was on a voluntary basis. After the pilot application with an athlete, necessary corrections were applied, and the final shape was given for use in the study. Interviews with athletes in the VMBR + VM and VRBI groups, which took approximately 10 min, were recorded for analysis. Interviews were conducted with a total of six athletes; three from the VMBR + VM group and three from the VRBI group. After it was understood that the data had reached the saturation level, the data collection was terminated.

### Process

The MIQ-R was filled by all participants to determine the imagery skill levels of athletes. In addition, 10 warm-up shots and 5 actual shots were made to determine the pre-test scores of the athletes. These shots were repeated on three different days of the week. The pretest score was created by taking the average of 15 actual shots (3 days ^∗^ 5 shots). Then all participants were randomly divided into VMBR + VM group, VRBI group, and Control groups.

**FIGURE 3:**
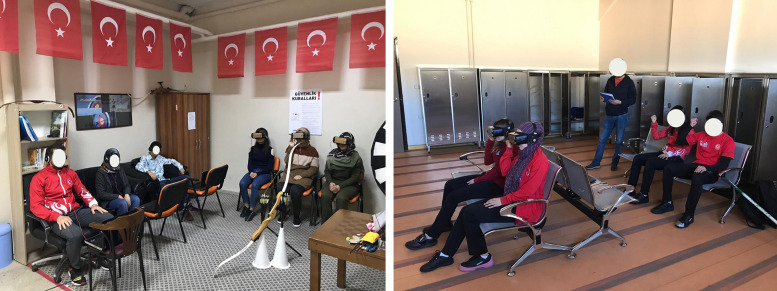
Watching performance videos.

The VMBR + VM group performed progressive muscle relaxation exercise with 2D video guidance. At the end of the relaxation exercise, VM was done by watching the previously prepared successful 2D performance video from the laptop (Figure 3). Then the athlete imagined that he performed this performance within the framework of the imagery script. At the end of the imagery, performed the same performance that he had seen in the VM, performing 10 warm-up shots and 5 actual shots in the real training area. The daily performance score was recorded in the personal file of the athlete, taking the average of the 5 actual shots made.

Virtual reality based imagery group did progressive muscle relaxation exercise with 3D video guidance. Then, 3D-VM was done by watching the previously prepared successful shot video in 3D environment with VR glasses (Figure 3). After the imagery training was completed, the athletes were asked to take a successful shot similar to the shot in the VRBI training scenario. After 10 shots were made for practice, 5 real shots were made. All the shots made by the athletes in the VMBR + VM and VRBI groups were scored by the experts. In the control group, after the fun videos about the branch were watched, 10 warm-up and 5 main shots were made to record their daily performances. Imagery trainings, including warm-up trainings, were made for each branch, for approximately 2 h a day, 3 days a week, and for a total of 4 weeks. The weekly average of the daily performance scores obtained was taken. The MIQ-R applied at the beginning of the study was reapplied to the athletes at the end of 4 weeks and the changes in the athletes were observed longitudinally. The quantitative data obtained after the application phase of the research has been analyzed. Accordingly, one-to-one interviews were conducted with the athletes within the framework of the interview form prepared by the researcher. The data obtained was recorded on the voice recorder.

### Data Analysis

Normal distribution of continuous variables was evaluated with the Shapiro–Wilk test, Skewness and Kurtosis values. Since the number of participants in the study group is less than 35, Shapiro–Wilk normality test was used (Shapiro, 1985). The values obtained are presented in Table 2.

**TABLE 2:** Normality analysis results of the participants.

	**Group**	**Measurement**	***n***	**x̄**	**ss**	**S**	**K**	**SW**
Shot performance	Control	Pre-test	11	5,74	1,91	−0,421	−0,905	0,575
		(1) Week	11	5,90	1,89	−0,350	−1,341	0,209
		(2) Week	11	5,60	2,37	−0,247	−0,535	0,837
		(3) Week	11	5,95	2,14	0,109	−0,516	0,946
		(4) Week	11	5,90	2,18	−0,144	−0,409	0,991
	VMBR + VM	Pre-test	11	5,21	1,02	0,933	0,528	0,102
		(1) Week	11	4,79	1,92	0,273	−1,197	0,408
		(2) Week	11	5,70	1,41	0,761	−0,797	0,122
		(3) Week	11	6,43	1,45	0,869	0,219	0,526
		(4) Week	11	6,60	1,47	0,932	0,138	0,124
	VRBI	Pre-test	12	4,68	0,98	0,296	−0,997	0,657
		(1) Week	12	4,22	2,17	0,882	0,117	0,157
		(2) Week	12	6,60	1,53	0,498	−0,585	0,567
		(3) Week	12	6,98	1,46	0,087	−1,165	0,383
		(4) Week	12	7,33	1,19	0,442	0,153	0,353
MIQ-R	VMBR + VM	Pre-test	11	3,97	0,63	0,183	−0,973	0,747
		Post-test	11	4,17	0,54	0,422	−1,727	0,052
	VMBR + VM	Pre-test	11	3,97	0,76	−0,907	−0,258	0,161
		Post-test	11	5,27	0,67	−0,318	0,190	0,987
	VRBI	Pre-test	12	3,57	1,03	−0,459	0,560	0,894
		Post-test	12	5,04	0,66	−0,030	-0,543	0,780

*S, skewness; K, kurtosis; SW, Shapiro–Wilk(p).*

George and Mallery (2016) state that the skewness and kurtosis coefficients should be between −2 and +2 values accepted for social sciences, according to the 5% significance level, in order for the data set to be suitable for normal distribution.

In addition, Levene test was performed for the homogeneity of variances, another parametric test assumption (Pasin et al., 2016). The values obtained are presented in Table 3.

**TABLE 3:** Variance homogeneity (Levene) test results applied to GMDP + VM, SGTI, and control group scores obtained from repeated measurements.

	**Measurement**	***n***	**df1**	**df2**	***F***	***p***
Shot performance	Pre-test	34	2	31	3,076	0,057
	(1) Week	34	2	31	0,003	0,997
	(2) Week	34	2	31	1,496	0,240
	(3) Week	34	2	31	0,844	0,440
	(4) Week	34	2	31	2,303	0,117
MIQ-R	Pre-test	34	2	31	0,773	0,470
	Post-test	34	2	31	0,016	0,984

As a result of the tests, it was seen that the data met the parametric test assumptions. Mixed Measure Two Way ANOVA was performed to determine the effect of the imagery training program application on the dependent variable (shot performance, imagery skills). Mauchly’s test was applied to identify the data sphericity. When the sphericity assumption was violated, the Greenhouse–Geisser correction was used. A significance level of *p* ≤ 0.05 was taken for all results reported. *Post hoc* comparisons were conducted through Bonferroni significant difference tests. Qualitative data obtained in the study was analyzed with the content analysis method. SPSS was used for the analysis of quantitative data, and NVIVO programs were used for the analysis of qualitative data.

## Results

This section includes detailed results of the analyzes made in line with the hypotheses of the research. The main purpose of the research is to examine whether the newly prepared VRBI program is more advantageous than the widely used VMBR + VM today.

Weekly shot averages and standard deviation values of the athletes participating in the research are given in Table 4.

**TABLE 4:** Weekly average and standard deviation values of the shot performance scores of the athletes participating in the research by groups.

**Groups**	**Pre-test**		**(1) Week**		**(2) Week**		**(3) Week**		**(4) Week**	
	**x̄**	**ss**	**x̄**	**ss**	**x̄**	**Ss**	**x̄**	**ss**	**x̄**	**ss**
Control	5,06	1,36	4,97	1,55	5,00	1,56	5,35	1,47	5,37	1,44
VMBR + VM	5,21	1,02	4,79	1,92	5,70	1,41	6,43	1,45	6,60	1,47
VRBI	4,68	0,98	4,22	2,17	6,60	1,53	6,98	1,46	7,33	1,19

As observed in Table 4, weekly changes of the athletes in the control group are not significant; however, there is an increase in the weekly shot performance averages of the VMBR + VM and VRBI groups.

The results of (3 × 5) ANOVA regarding whether or not the weekly changes in the shot performances of athletes exposed to three different experimental procedures show a significant difference are given in Table 5.

**TABLE 5:** ANOVA results of the shot performance scores of the athletes participating in the research.

**Source of variance**	**SoS**	**df**	**MS**	***F***	***p***
Between subject	299,488	33			
Group	19,949	2	9,974	1,106	0,344
Error	279,539	31	9,017		
Within subject	189,009	97,762			
Measurement (weekly)	81,743	2,875	28,429	36,590	0,000*
Measurement *Group	38,012	5,751	6,610	8,508	0,000*
Error	69,254	89,136	0,777		

*p < 0,05; SoS, sum of squares; MS, mean square.

When Table 5 is examined, it is determined that there is no significant difference between the VMBR + VM, VRBI, and the athletes in the control group in terms of their scores in shot performance [*F*(2,31) = 1.106, *p* > 0.05]. When the athletes included in the research are compared regardless of which group they are, the difference between the pre-test, week 1, week 2, week 3, and week 4 performance scores was found to be significant [*F*(2.875,89.136) = 36,590, *p* < 0.05]. This result shows that the performance scores of athletes increased in the process. In order to see the effects of the process on the score differences between the groups, the measurement ^∗^ group joint effect of the test results of the shot scores of the athletes was examined and this value was found to be significant [*F*(5.751,89.136) = 8,508, *p* < 0.05]. Therefore, it was determined that the imagery training applied had an impact on the athlete’s performance scores.

Multiple comparison test results to determine which intergroups differ according to the process are given in Table 6.

**TABLE 6:** Multiple comparison test results for shot performance points by group and measurement time.

**Measurement time**	**Group**		**Mean difference (I–J)**	***p***
Pre-test-(1) week	Control	VMBR + VM	0,333	0,511
		VRBI	0,367	0,463
	VMBR + VM	VRBI	0,031	0,950
Pre-test-(2) week	Control	VMBR + VM	−0,536	0,245
		VRBI	−1,971	0,000*
	VMBR + VM	VRBI	−1,434	0,003*
Pre-test-(3) week	Control	VMBR + VM	−0,925	0,030*
		VRBI	−2,016	0,000*
	VMBR + VM	VRBI	−1,090	0,010*
Pre-test-(4) week	Control	VMBR + VM	−1,068	0,010*
		VRBI	−2,336	0,000*
	VMBR + VM	VRBI	−1,268	0,002*

*p < 0,017.

When Table 6 is examined, it is observed that there is no difference between the pre-test and 1st week shot performance scores between the groups. In the 2nd week, significant differences were observed between the VRBI group and VMBR + VM and the control groups, while no significant differences were found between VMBR + VM and the control group. When the measurement scores made at the end of the 3rd and 4th weeks were compared with the pre-test results, it was observed that there was a significant difference between the VMBR + VM, VRBI, and control groups. When the findings were examined, it was observed that the shot performances of the VRBI group started to rise earlier than those in the VMBR + VM group did. In addition, it was observed that the shot performances of the athletes in the VRBI group were better than the athletes in the VMBR + VM group, as well those in as the control group in the 2nd, 3rd, and 4th weeks. As a result, it can be said that VRBI training is more effective than VMBR + VM in shot performance and adaptation.

**FIGURE 4:**
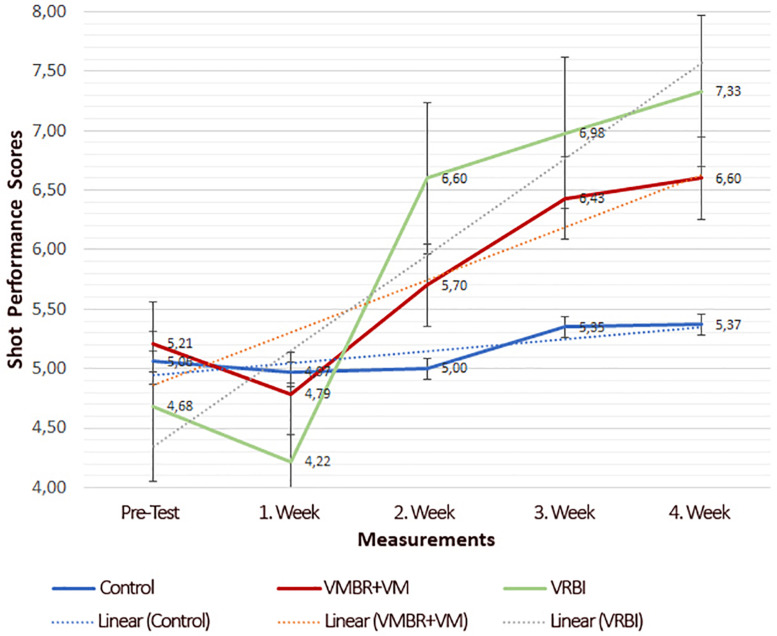
Weekly result graphic of shot performance score by groups.

In Figure 4, the changes in the shot performances of the groups during the experimental process are shown graphically. When the figure is analyzed, it is seen in the measurements made at the end of the first week that the athletes in the VMBR + VM and VRBI groups experienced serious decreases in their shot performances, and this decline was replaced by an increase. No significant difference was observed in the shot performances of the athletes in the control group.

When Table 7 is analyzed, it is seen that the pre-test scores of the athletes in the control and experimental groups from the imagery skills are close to each other, and that there are differences in the post-test scores.

**TABLE 7:** Weekly average and standard deviation values of the imagery skill points of the athletes participating in the study according to the groups.

**Groups**	**Pre-test**		**Post- test**	
	**x̄**	**ss**	**x̄**	**ss**
Control	3,97	0,63	4,17	0,54
VMBR + VM	3,97	0,76	5,27	0,67
VRBI	3,57	1,03	5,04	0,66

When Table 8 is analyzed, it is seen that there was no significant difference between the VMBR + VM, VRBI, and control group athletes in terms of imagery skill scores [*F*(2,31) = 2,643, *p* > 0.05]. Regardless of the group of athletes included in the study, the difference between the imagery pre-test and post-test scores was found to be significant [*F*(1,31) = 35,771, *p* < 0.05]. This result shows that the intervention of this process has increased the imagery skill scores of athletes. It is observed that the results of the imagery skill scores of the athletes have a significant effect on the measurement ^∗^ group [*F*(2,31) = 5,833, *p* < 0.05]. As a result of this finding, it can be said that the types of imagery training caused a differentiation between the groups in terms of imagery skill scores.

**TABLE 8:** ANOVA results of the imagery skill points of the athletes participating in the research.

**Source of variance**	**SoS**	**df**	**MS**	***F***	***p***
Between subject	23,123	33			
Group	3,369	2	1,684	2,643	0,087
Error	19,754	31	0,637		
Within subject	36,165	34			
Measurement (weekly)	16,493	1	16,493	35,771	0,000*
Measurement *Group	5,379	2	2,689	5,833	0,007*
Error	14,293	31	0,461		

*p < 0,05; SoS, sum of squares; MS, mean square.

Multiple comparison results made in order to understand between which groups this difference is are given in Table 9.

**TABLE 9:** Multiple comparison test results of imagery skill pretest and post-test scores by groups.

	**Group**		**Mean difference (I–J)**	***p***
		
Imagery skills	Control	VMBR + VM	−1,102	0,011*
		VRBI	−1,275	0,003*
	VMBR + VM	VRBI	−0,173	0,668

*p < 0,017.

When Table 9 is examined, it is seen that according to the pre-test/post-test results, the imagery skills of the VMBR + VM and VRBI groups improved significantly and positively. At the end of the process, it was observed that there were no significant difference between the VMBR + VM and VRBI groups in terms of imagery skill scores.

**FIGURE 5:**
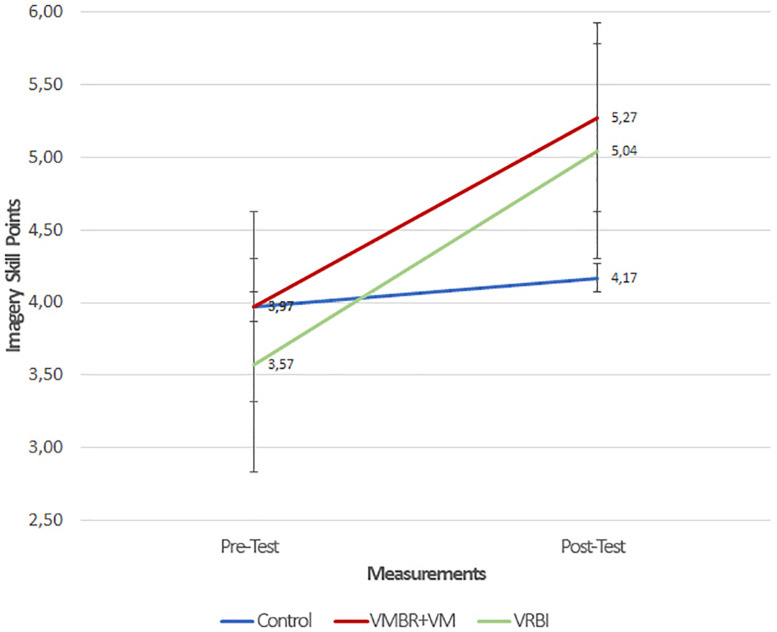
Pre-test post-test result graphic of imagery skill score by groups.

When Figure 5 is examined, we can see there is an increase in the imagery skill scores of the VMBR + VM and VRBI groups in the pre-test/post-test results, while no significant increase was observed in the control group.

Content analysis results for determining the VRBI experiences of athletes are given in Table 10.

**TABLE 10:** Analysis results of VRBI experiences of athletes.

**Theme**	**Code**	f*
Emotional and mental responses	Stress	1
	Prejudice	4
	Concentration problem	2
	Excitement	2
	Frustration	1
Getting used to and believing	Adaptation	1
	Self-confidence	2
	Changing perspective	3
Successful imagery	Entering virtual reality environment	3
	Increased concentration	3
	Total	22

*Athletes stated more than one opinion.

The data obtained as a result of the interviews with the athletes are shown in Table 10. When the data in the table and the athletes’ interviews were examined, the VRBI training program initially caused negative feelings such as stress, prejudice and concentration problems for the athletes. These negative emotions were replaced by positive emotions such as self-confidence and adaptation with the progress of the process. At the end of the process, it can be seen that the athletes could now immerse themselves in the VR environment and use this training method to increase their concentration before the competition.

Examples of answers given by athletes are given below.

A4: Since I used it for the first time in my life, I said to myself what effect it would have at first. I thought it would have no effect. I didn’t feel any effect in the first weeks because I started this process biased.

A6: Initially, I thought this was ridiculous and would have no effect on my performance.

A5: I realized that when I started to get used to mental training and when I did it correctly, there was improvement and progress in my shots. This led me to begin to believe this workout, which I had initially found ridiculous.

The quantitative data obtained from the study was analyzed and it was observed that the shot performance of the athletes in VMBR + VM and VRBI decreased in the first weeks. Content analysis results for determining the cause of this decline are given in Table 11.

**TABLE 11:** Analysis results related to the causes of performance losses in the first weeks.

**Theme**	**Code**	f*
Negative emotions	Prejudice	3
	Stress	3
Changing habits	New training model	3
	Total	9

*Athletes stated more than one opinion.

When Table 11 is analyzed, it is seen that the reasons for the loss of performance in the first weeks of athletes were due to prejudice, stress and a new application, and this caused the athletes to change their habits.

Examples of answers given by athletes are given below.

A1: Initially, I thought this was ridiculous, so I had a bias.

A2: It didn’t make any sense to me when I first put on the virtual reality glasses. It caused me to get worse rather than improve my performance. Because thinking that we should do a task given to us in the first training made me stressful. Somebody comes and makes you do something, and in return, it measures your performance. Due to this pressure, my shot performance decreased and my stress level increased.

## Discussion

The aim of this study was to examine the effect of VRBI training program on athletes’ shot performance and imagery skills, and to compare it with VMBR + VM, which is the popular approach used in imagery training today.

### Main Findings

The findings show that the new imagery model VRBI training program developed by the researcher gives more positive results in terms of shot performance when compared to the most popular imagery training program, VMBR + VM. In addition, it is seen that the athletes in the experimental group (VRBI and VMBR + VM) experienced performance loss in the first week of the research due to various reasons. Another important result of the study was that the athletes in the VRBI group adapted to the process faster and as a result experienced a rapid increase in performance. Finally, it was observed that the imagery skills of the athletes in both experimental groups increased during the research.

### VR Technology, Imagery and Performance Improvement

Virtual reality is widely used in this field as it provides the opportunity to include or exclude the desired variables in the process and create replicable simulations to determine the performance of the athlete (Bideau et al., 2010). Thanks to the developing technology, real competition areas can be created in the VR environment. The athlete, who is in a realistic VR environment, act physical and psychological reactions (sweating, anxiety, etc.) as in the real competition (Stinson and Bowman, 2014). In this research, real competition environment is tried to be simulated in virtual environment. The improvement of the shot skills of the athletes who train in the virtual environment is one of the important findings of the research. In the literature, it is seen that VR training programs designed to improve the skills of athletes are effective (Neumann et al., 2018; Michalski et al., 2019).

Virtual reality has the potential to include PETTLEP components in the imagery training. The inclusion of PETTLEP components such as physical, environment, task, time, learn, emotion, and perspective in the imagery training enhances the effect of imagery (Smith et al., 2008, 2019; Wright and Smith, 2009; Battaglia et al., 2014; Anuar et al., 2018). In the mechanism underlying the VRBI program, all the components in the PETTLEP model are included in the process to maximize the effect of imagery. For this purpose, a new 3D model has been designed to include all the sensory organs in the imagery process and make the athlete feel as if the competition is happening in real life at that moment.

When creating vivid and clear images in the mind, more kinesthetic emotions are required than visual details (Smith et al., 2001). Thanks to the VRBI program, athletes can also activate their kinesthetic feelings by physically doing the same motion imagined, as they can move freely at the time of imagery and 3D VM. The kinesthetic ability associated with visual ability is crucial to improving motor performance (Ille and Cadopi, 1999). Pelgrims et al. (2009) argue that motor imagery is often more affected by action-related kinesthetic restrictions. Thanks to VRBI, this restriction has been eliminated.

The most important difference of the VRBI training model to the imagery training done so far is that the entire imaging process is done in 3D environment. In particular, the findings have shown that making VM in a 3D environment has important contributions. Particularly, athletes in the VRBI group reported that they were putting themselves in the place of the successful shooter in the video while modeling the video in a VR environment, thus increasing their self-confidence. In addition, athletes reported that they felt as if they had repeatedly done the same movement physically. It is the common idea of all the imagery theories that the imagery made close to reality has the highest impact on athletes. Another extension of the PETTLEP model is VM, which is of great interest in neuroscience (Holmes and Calmels, 2008; Battaglia et al., 2014). Watching themselves or others can not only improve the performance of the athlete, but also has a positive effect on psychological skills such as self-efficacy and self-regulation (Murphy, 2012). Nowadays, imagery and VM exercises are used in combination with each other to increase the psychological and physical performances of athletes. Research has shown that the combination of imagery and VM significantly improves performance (Smith et al., 2007; Wright and Smith, 2009; Battaglia et al., 2014; Buck et al., 2016).

The reasons for the positive effect of VM on athletes’ performances have been studied in the research field for many years. Cognitively, an athlete observes the skill and encodes it symbolically and then uses this encrypted information as a guide for future action (SooHoo et al., 2004). This situation is based on symbolic learning theory.

The positive contribution of VM to athletes’ performance can be explained neurologically with mirror neurons. Mirror neurons are neurons that activate when watching a movement (Demir and Gergerlioğlu, 2012). Mirror neurons differ from superior temporal sulcus neurons that only control visual processes, since the motor and visual responses are located in the same neuron (Herrington et al., 2011). It is thought that the superior temporal sulcus, activated by the observation of the biological movement, provides visual information to the mirror neuron system (Lange and Lappe, 2006; Molenberghs et al., 2010). The VR environment facilitates the activation of mirror neurons (Merians et al., 2006). Thus, it is easier and more effective to imitate the movements in a video when it is watched in a VR environment. In 3D VM in VRBI, it is thought that utilizing the 3D VR environment increases the activation of mirror neurons and causes the athletes in this group to perform more highly with their shot to outperform the athletes in the VMBR + VM group.

Whether there are differences in brain activation during actual and virtual actions is controversial. An fMRI study showed that the mirror neuron system was strongly activated by seeing both human and virtual (robotic) actions, without any significant difference between the two states (Gazzola et al., 2007). This shows that the region related to the movement in the brain may be more important than the way the action is performed. Recent research has shown that corticoline excitability increases during targeted VR mirror exercise when compared to true mirror exercise (Aziz-Zadeh et al., 2004; Kang et al., 2012).

In order to investigate the effect of the VR environment on mirror neurons, stroke patients were applied a virtual hand movement rehabilitation program in the VR environment (August et al., 2006). The findings obtained showed that the areas such as primary motor cortex, dorsal premotor cortex, where mirror neurons are found, were more active in the exercise program group who performed this in a VR environment. The findings obtained support the study. It is thought that the shot performances of the athletes in the VRBI group were better than the athletes in the VMBR + VM group, due to both the symbolic learning theory and the activation of mirror neurons.

### Performance Loss in the First Weeks

An important point in the findings obtained is the decrease in shot performance of the athletes in the VRBI and VMBR + VM group during the first week of their imagery training. According to the qualitative research results regarding the causes of this decline, it was understood that the athletes in both the VMBR + VM group and the VRBI group developed negative emotions such as stress, excitement, and concentration problems at the beginning of the study. It is seen that athletes who had not received any of the psychological skill training professionally have developed negative feelings toward this new situation. Since high anxiety prevents the creation of successful images in the mind, the athlete cannot reach maximum performance (Suinn, 1972a; Morris et al., 2005). In addition, another result from the qualitative data is that athletes are biased toward VRBI and VMBR + VM training programs. The athletes thought that these exercises would not be useful because they had to change their habits with the new training program, and did not have enough experience and knowledge about the process. This situation adversely affected the shot performances. Whatever the reason, sports psychology suggests various psychological skills training in order to overcome the negative emotions that can occur in the athlete. Imagery, the most important of this skill training, can help athletes get rid of their negative emotions. In the literature review conducted in this area, it has been proved that the imagery exercises have benefits such as controlling emotions (Koehn et al., 2006; Jeong, 2012; Carter, 2013; Bayköse, 2014), reducing anxiety (Kolayis, 2002; Erdoğan, 2009; Vurgun, 2010; Akkarpat, 2014; Güvendi, 2015), improving concentration (Uludağ et al., 2016; Tekin, 2018) and gaining self-confidence (Callow and Waters, 2005; Akkarpat, 2014). In the later days of the research, it is thought that athletes get rid of these negative emotions both by adapting to the process and by continuing their imagery training. It was observed that the shot performances of the athletes, who focused on their imagery training and performances by getting rid of negative emotions, increased significantly.

### VR Technology Accelerates Adaptation to the Process

Another important finding obtained from the research is that for the athletes in the VRBI group, the increase in their shot performance and adaptation to the process was faster than the athletes in the VMBR + VM. In order to better understand why athletes in the VRBI group adapted faster to the process and increased their performance faster than the VMBR + VM group, the weekly changes in the shot performances of the groups are examined. An important result is the adaptation time of the athletes to the imagery training thanks to the VRBI intervention. In this way, the effect of the imagery training is seen in a shorter time. This effect occurs about a week (three workouts) before than VMBR + VM group. The results show that athletes in the VRBI intervention group adapt more quickly to their imagery training and that their imagery skills develop faster.

It is believed that isolation of the athletes in the VRBI group from the outside world through VR glasses and headphones and the inclusion of kinesthetic, visual and auditory senses in the process effectively helped to shorten the adaptation process in athletes. Information obtained from interviews with athletes confirms this idea.

In addition, using the 3D environment in the imagery process alleviates the cognitive load of the individual (Yiasemidou et al., 2017). This enables the athletes to adapt to the imagery training more quickly and to show a rapid performance improvement. In this study on rowing athletes, Parton and Neumann (2019) applied a VR protocol training program and examined the effect of this exercise program on the performance and motivation of athletes. According to the findings obtained in the study, it was found that VR-based training program was beneficial in increasing the motivation and performance of athletes. The findings are in line with the findings of this study. In this study, the effects of supporting the use of motor imagery with the VR program on corticomotor excitability were investigated (Im et al., 2016). Participants, consisting of 15 stroke patients and 15 healthy individuals were randomly assigned to control, motor imagery, SG-guided motor imagery and SG-guided motor imagery groups with task variability. According to the findings obtained as a result of the application, corticomotor excitability increased in all the subject groups. In addition, subjects in the SG guided imagery group were found to have greater central nervous system responses (motor arousal potential) given stimulus than subjects in the imagery group only. The findings clearly show the positive contribution of the VRBI training to the imagery skills of athletes, and these results overlap with the literature.

### Improvement Imagery Skills

In addition to the improvement of the shot performances of the athletes in the VRBI and VRBI + VM groups, it was also found that the imagery skills showed a significant improvement when compared to the control group. When the findings obtained are examined, in terms of imagery skills there are significant differences between VMBR + VM and VRBI groups and control groups (VMBR + VM – control, VRBI – control). Another noteworthy situation here is the increase in the imagery skills of all groups, including the control group. Although there is no statistically significant increase, it is thought that the reason for this increase in the control group is that MIQ-R is an applied scale. Findings from neurophysiological and behavioral neuroimaging studies show that the application of motion has the potential to affect the imagery ability (Williams et al., 2011). In studies conducted using neuroimaging, it is shown that performing motion contributes to motor imagery by improving neural activity (Decety, 1996; Fadiga et al., 1999; Buccino et al., 2001; Ehrsson et al., 2003; Clark et al., 2004).

The main hypothesis of the study was that VRBI training would contribute more positively to athletes’ shot performance and imagery skills than VMBR + VM training. To test this hypothesis, the shot performances of the groups and the changes in their imagery skills were analyzed. In the findings obtained as a result of the 4-week study, it was seen that VMBR + VM significantly improved the shot performance and imagery skills of the athletes when compared to the control group. However, VRBI training seems to give much better results when compared to VMBR + VM.

Virtual reality based imagery includes all the sensory organs of the athlete, which uses the PETTLEP components, to the mental preparation process. In order to do this, athletes practice the imagery in a 3D environment. Training in a 3D environment not only allows the athletes to break away from the outside world, which negatively affects the imagery process, but also makes it easier for them to give their whole perception to the process of imagery. VRBI training program provides an excellent infrastructure for the PETTLEP model. The environment, which is one of the components in the PETTLEP model, is presented to the athlete as very close to reality thanks to VR. It helps the athlete create the competition area with more vivid and clearer images in his mind. At the moment of sports imagery and VM, instead of designing the competition area in his mind, he can give all his concentration to the movement. Thus, the athlete can reduce his cognitive load in his mind and give his full focus to the imagery. The timing component is also used effectively in imagery, thanks to the VRBI program. Researchers have found that doing the imagery at the actual speed of the movement will increase the quality of the imagery (Holmes and Collins, 2001). Thanks to the VRBI program, the performance in the shot mission is shown at its real speed. In different studies, it has been suggested to use an internal perspective in imagery exercises to develop psychological skills such as motivation, concentration and focus (Mahoney and Avener, 1977). The reason for this is that the person who makes the imagery dreams that he is the person who makes the movement. Since the VRBI training program takes place in a 3D environment, the athlete is provided with an internal perspective through VM and imagery processes, and this is done in the most realistic way possible.

The results obtained show that the VRBI program gave more positive results both in terms of performance and imagery skills when compared to the control group and VMBR + VM group. VR technology provided a great advantage in terms of the feeling of the imagery athlete in that environment and self-control of the process.

### Limitations and Future Directions

There are a few limitations to the present study that should be addressed in future work. Since the athletes from three different branches participated in the research, the most important limitation of this study is that the expertise levels of the athletes in these branches are not exactly the same. Although the criteria for participants to be elite athletes are set in order to overcome this situation, the selection of athletes from the same branch may be preferred in similar studies to be conducted in the next. Another limitation of the study is that the participants are partially passive in the VR environment (can only move their heads) and cannot make hand and arm movements within the virtual world. A new study can be done by creating a fully interactive VR environment in future studies. In the study, a total of 15 shots, 5 shots per day and 3 days per week, were made and pre-test scores were created. Although the same method was used to create a pre-test score in similar studies (García-Herrero et al., 2016; Kehoe and Rice, 2016; Gray, 2017; Robin et al., 2019), more shots could be taken to avoid measurement errors. However, an important limitation here is that ice varies continuously in the curling branch. In the shots more than 15 shots (10 warm-up – 5 actual shots), the structure of the ice changes and the stone goes faster and more rotating. Therefore, the number of daily shots was limited to 15. As a result of the 4-week training program, it is seen that the performance increase continues in the experimental groups. By conducting longer studies, it can be seen how many weeks this linear increase will end. Moreover, in order to explain the effectiveness of this developed SGTI training program concretely, it should be supported with neurophysiological findings (EEG, fNIRS, fMRI, etc.).

## Data Availability Statement

All datasets presented in this study are included in the article/supplementary material.

## Ethics Statement

This research was designed in accordance with the Helsinki Declaration and was approved by the Atatürk University Faculty of Medicine Ethics Committee (No: B.30.2.ATA.0.01.00/218, Date: 19.09.2009). All participants read and signed the voluntary consent form before starting the research.

## Author Contributions

DB and SE designed the experiments, commented on the data, wrote the original draft, and reviewed, edited, and prepared the article and approved the latest version. DB collected the data and carried out statistical analysis. Both authors contributed to the article and approved the submitted version.

## Conflict of Interest

The authors declare that the research was conducted in the absence of any commercial or financial relationships that could be construed as a potential conflict of interest.
